# Beauvericin and Enniatins: In Vitro Intestinal Effects

**DOI:** 10.3390/toxins12110686

**Published:** 2020-10-29

**Authors:** Alessia Bertero, Paola Fossati, Doriana Eurosia Angela Tedesco, Francesca Caloni

**Affiliations:** Department of Environmental Science and Policy (ESP), Università degli Studi di Milano, Via Celoria 10, 20133 Milan, Italy; alessia.bertero@unimi.it (A.B.); paola.fossati@unimi.it (P.F.); doriana.tedesco@unimi.it (D.E.A.T.)

**Keywords:** *Fusarium* mycotoxins, beauvericin, enniatins, molds, in vitro intestinal models, toxicology, species-specificity

## Abstract

Food and feed contamination by emerging mycotoxins beauvericin and enniatins is a worldwide health problem and a matter of great concern nowadays, and data on their toxicological behavior are still scarce. As ingestion is the major route of exposure to mycotoxins in food and feed, the gastrointestinal tract represents the first barrier encountered by these natural contaminants and the first structure that could be affected by their potential detrimental effects. In order to perform a complete and reliable toxicological evaluation, this fundamental site cannot be disregarded. Several in vitro intestinal models able to recreate the different traits of the intestinal environment have been applied to investigate the various aspects related to the intestinal toxicity of emerging mycotoxins. This review aims to depict an overall and comprehensive representation of the in vitro intestinal effects of beauvericin and enniatins in humans from a species-specific perspective. Moreover, information on the occurrence in food and feed and notions on the regulatory aspects will be provided.

## 1. Introduction

Mycotoxins are secondary metabolites produced by different molds that frequently contaminate food and feed [1] and have potential adverse effects on human and animal health [2]. Mold growth and mycotoxin synthesis can occur at any stage of production and supply chains, from the field to the plate [3] and under the influence of many complex and interconnected environmental factors (i.e., nitrogen, carbon, water, pH, etc.) [4]. These are difficult to control and manipulate in order to avoid contaminations, even if good agricultural practices are applied. Therefore, mycotoxins mostly represent an unforeseeable and unpreventable issue for human and animal health, even more so since detoxification procedures are often impractical and unsatisfactory [5,6,7].

Emerging mycotoxins, namely toxic fungal metabolites, are “neither routinely determined, nor legislatively regulated; however, the evidence of their incidence is rapidly increasing” [8]. These are currently of constant interest and concern. On the one hand, more sophisticated analysis methods able to simultaneously detect different mycotoxins (including emerging mycotoxins) and provide more comprehensive exposure information have been developed, from sample cleanup to chromatographic techniques (i.e., high-performance liquid chromatography–mass spectrometry) [9,10]. On the other hand, awareness about this topic has increased because of the challenge posed by climate change in terms of food and feed safety [11,12]. Indeed, high mycotoxin contamination has often been associated with unusual weather [6], even if, to date, more information on the effects of climate on mold germination, growth and mycotoxin production is necessary to evaluate the actual entity of this relatively recent phenomenon [13]. Furthermore, it cannot be ignored that toxicological data, particularly on emerging mycotoxins, are still scarce. There is a strong need for a deeper comprehension of the toxicological potential of these particular compounds [14,15,16]. This is also relevant from a mechanistic perspective and particularly in the long term and regarding coexposures [15,17].

Since ingestion is the major route of exposure to mycotoxins in food and feed [18,19], the gastrointestinal tract is the first organ encountered by these compounds. Thus, besides acting as a barrier against mycotoxin penetration into the organism, it is also the first structure affected by their toxic actions [3,12], and a comprehensive toxicological evaluation cannot disregard this fundamental target [20]. Different intertwined elements, namely the mechanical (intestinal epithelium), chemical (i.e., gastric acids, bile, digestive enzymes, mucopolysaccharides, etc.), immune (intestinal immune system) and biological (intestinal microbiota) components, constitute the intestinal barrier and can all be affected by mycotoxins [3,21]. Various in vitro intestinal models have been used to investigate the toxicological actions of these natural contaminants, particularly cellular models and other systems able to recreate the different aspects of the intestinal environment.

In this scenario, this review aims to provide insight into the in vitro intestinal effects of the major emerging mycotoxins enniatins (ENNs) and beauvericin (BEA), highlight the diverse approaches adopted with different in vitro models (human and species-specific [22]) and provide information on the occurrence in food and feed and notions on legislative aspects.

## 2. Beauvericin

Beauvericin (BEA) is an emerging mycotoxin synthesized as a secondary metabolite by toxigenic molds included in the *Fusarium* genus and, to a lesser extent, in other genera such as *Beauveria* [23] and *Isaria* [24]. This compound can occur in a wide range of food and feed products worldwide [10,25,26,27,28,29,30,31,32,33], from cereal grains [15] and dried fruits [34] to eggs [35], representing a critical risk factor for human, animal and environmental health [32,33]. In fact, high prevalence and concentrations (up to hundreds of mg/kg) have been reported for this mycotoxin in food and feedstuffs [32]. Notably, low micromolar concentrations of BEA have also been found to exert cytotoxic effects against different mammalian cell lines in vitro, for instance: Caco-2 cells [36,37], KB-3-1 cells [38], CHO-K1 cells [39,40,41,42], human dendritic cells and macrophages [43], human acute lymphoblastic leukemia cells [44], human lymphoblastoid Jurkat T cells [45,46], hepatocellular carcinoma line Hep G2 [47,48], fibroblast-like fetal lung MRC-5 cells [47], porcine kidney PK15 cells [49] and IPEC-J2 cells [50], among others. Indeed, BEA is a potent bioactive compound [15] possessing antibacterial [51,52,53,54,55], antiviral [56], antifungal [57,58], insecticidal [59,60,61,62,63], anthelmintic [64], phytotoxic [65], endocrine disruptor [37,66,67] and anticancer activities [68,69,70].

Regarding its structure, BEA is a cyclic hexadepsipeptide that is chemically related to ENNs (it is included in the ENN antibiotic family), identified by the alternate presence of three D-α-hydroxy-isovaleryl and three aromatic N-methyl-phenylalanyl groups [23]. Owing to its peculiar configuration, BEA (as well as ENNs) may act as a nucleophile because of the presence of free electron pairs that can establish chemical bonds (ion–dipole) with ion groups [2] to form complexes with several compounds, mainly metallic cations (but also other small charged and neutral molecules), and carry them across the lipophilic phase of the cellular membranes [70]. Moreover, these BEA complexes can aggregate into clusters, leading to the formation of channels into the lipid bilayer of the plasmalemma [2] that allow the passage of ions determining the perturbation of physiological extracellular and intracellular ion concentrations [33]. BEA exerts its toxicological effects through different mechanisms, which are, generally speaking, largely related to its ionophoric properties. These include: disruption of the cell cycle progression (cell cycle blockage) [36,41,45,71], apoptosis induction (i.e., through the upregulation of the mitochondrial release of cytochrome c with the activation of the caspase 3 apoptotic cascade [44,46,71] and/or triggering the increase of the intracellular Ca^2+^ concentration, which determines the activation of Ca-dependent endonuclease leading to DNA fragmentation and cellular death [2]), induction of oxidative stress with ROS production [36,40,72], genotoxicity [36,41,45,73] (contrarily, Dornetshuber et al. [38] suggested that reactive oxygen species (ROS) and DNA damage are not prime causes in BEA-mediated toxic effects), immunotoxicity [43], mitochondrial damage [36,41,45,74], inhibition of the acyl-CoA cholesterol acyltransferase (ACAT) activity [75], interaction with ATP-binding cassette (ABC) transporters ABCB1 and ABCG2 [76] and inhibition of cytochrome P450 (CYP3A4/5, CYP2C19, CYP3A1/2) activities [77].

### 2.1. In Vitro Effects of BEA on Human Intestinal Models

#### 2.1.1. Cell Models

**Figure 1:**
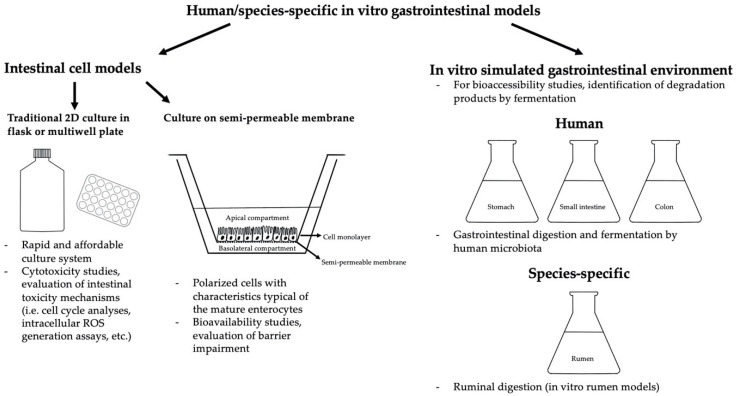
Characteristics of the major in vitro models applied to the investigation of the intestinal effects of mycotoxins.

Many studies on the toxicological effects of BEA have been performed using Caco-2 cell models (and other cell lines as well) evaluating bioavailability [78,79] cytotoxicity [36,37,78,79,80,81,82], intestinal barrier impairment [83], proinflammatory cytokine release [83], oxidative stress induction [36], DNA damage [36], cell cycle and mitochondrial alterations [36] (Table 1). Two major culture systems are usually applied, namely the traditional 2D in vitro culture in flasks or multiwell plates and 3D cultures on semipermeable Transwell^®^ inserts, which allow the cells to differentiate into a polarized monolayer showing characteristics that are typical of the mature enterocytes (i.e., expression of an apical brush border, tight junctions, desmosomes, metabolic enzymes of the intestinal epithelium, etc.) [84], mimicking the structure of the intestinal tract with the delimitation of an apical (Ap, intestinal lumen) and a basolateral compartment (Bl, lymphovascular) (Figure 1).

Bioavailability studies on BEA have been carried out using Caco-2 cells grown in two-compartment transwell models [78,79]. Indeed, due to the correlation observed between human oral absorption data and the results obtained with Caco-2 cells [85], this model is considered a reliable method to evaluate passive xenobiotic absorption through the intestinal barrier. Prosperini et al. [79] investigated the transepithelial transport and bioavailability (Ap to Bl) of 1.5 and 3 μM BEA after 1–4 h, obtaining a transport profile at 4 h ranging from 54.3 ± 1.1% (1.5 μM BEA) to 50.1 ± 1.1% (3 μM BEA). The concentrations detected inside the cells were 0.50 ± 0.05 μM (33% of the 1.5 μM BEA added at the beginning of the experiment) and 0.44 ± 0.03 μM (14.6% of the 3 μM BEA added at the beginning of the experiment). These high values likely depend on the presence of amino acid groups in the lateral chain of the BEA molecule that may determine the internalization of the mycotoxin or bind some components of the cellular membrane. Similar transport profiles were found in another work that reported low bioaccessibility values for BEA (<50%) [80]. Another study [78] employed Caco-2 cells cocultured with RAW 264.7 cells (murine macrophages) in a two-compartment transwell model to evaluate the bioavailability of BEA and silibinin (a pharmacologically active compound extracted from *Silybum marianum* that has been evaluated for its potential hepatoprotective effect against mycotoxin toxicity [86]), finding 10.40 ± 3.89% of the mycotoxin added at T0 into the Ap medium, 10.36 ± 0.98% into the Caco-2 cells, 2.31 ± 0.24% into the Bl medium and 0.57 ± 0.03 into the Raw 264.7 cells. Based on these results, since the total amount of BEA recovered in the system was lower than the quantity added into the Ap compartment at the beginning of the experiment, Tran et al. hypothesized that BEA may have undergone significant metabolization. Moreover, in accordance with the results obtained by Prosperini et al. [79], a significant fraction of mycotoxin was found in the cellular matrix (mainly in the Caco-2 cells but also in the RAW 264.7), thus confirming the supposition of an interaction between BEA and the cellular membrane (data that were also in line with the in-silico-predicted lipophilicity of this mycotoxin by Tran and colleagues using ACD/Percepta [78]).

BEA can also exert cytotoxic effects on human intestinal cell models and many studies have evaluated this action using traditional culture systems (generally cells maintained in 96-well plates). Research performed on Caco-2 cells revealed a significant (*p* ≤ 0.01) decrease in the cell number after exposure to 1 μM BEA and a lethal cytotoxic action at 10 μM BEA [37]. On the contrary, no cytotoxic effects have been observed on this cell line in a concentration range up to 50 nM BEA [78]; however, Font et al. [80] observed that BEA cytotoxicity on Caco-2 cells was three- to four-fold lower than that exerted by ENNs. Another study [79] demonstrated a dose-dependent cytotoxic effect of BEA on both Caco-2 (IC_50_ at 24 h = 20.62 ± 6.9 μM; IC_50_ at 48 h = 12.75 ± 4.8 μM) and HT-29 (IC_50_ at 24 h = 15.00 ± 6.9 μM; IC_50_ at 48 h = 9.75 ± 4.4 μM) cells, with the latter showing a higher sensitivity towards this mycotoxin. BEA cytotoxicity was also evaluated using human gastric (N87) cells [81], which showed a relatively low sensitivity to this compound in comparison to the other cell lines, expressing IC_50_ = 27.5 ± 0.7 μM, while the Caco-2 cells displayed IC_50_ = 3.9 ± 0.7 μM [81]. Similar results have been obtained by Salim et al. [82], with Caco-2 IC_50_ values ranging from 4.87 ± 0.42 (24 h) to 3.16 ± 0.45 (72 h).

The ability of BEA to induce intestinal barrier impairment has also been investigated using Caco-2 cells cultured on inserts through the evaluation of transepithelial electrical resistance (TEER) [83]. Low doses (1.5 μM for 24 h) of BEA alone were not found to induce any effect on the TEER, whereas the association of 1.5 μM BEA + 1.5 μM fumonisin B1 determined a significant TEER decrease, indicating damage to the epithelial barrier. Similarly, BEA alone had no effect on the proinflammatory cytokine interleukin 8 (IL-8) release, but the association with deoxynivalenol (3.5 μM) induced a significant increase in IL-8 secretion into the culture medium [83], emphasizing the importance to perform a toxicological evaluation not only of mycotoxins alone but also in coexposure for an appropriate risk assessment since combined toxic effects can occur [87,88].

Mechanisms of BEA intestinal toxicity have also been investigated with the use of Caco-2 cells. Prosperini et al. [36] highlighted the role played by oxidative stress in the in vitro effects exerted by mycotoxins. Indeed, Caco-2 cells showed elevated production of oxidizing species after BEA exposure (reaching a two-fold higher reactive oxygen species (ROS) production after 120 min of treatment in comparison with the control), together with a dose-dependent increase of malondialdehyde (MDA) production (120% with 1.5 μM BEA and 207% with 3.0 μM BEA). A dose-dependent decrease in the intracellular glutathione (reduced form (GSH)) concentration was also observed (by 23% with 1.5 μM BEA and 31% with 3.0 μM BEA), while the glutathione disulfide (GSSG) levels started to increase (20%) only after exposure to the highest dose of mycotoxin (3.0 μM BEA). BEA also induced cell cycle disruption, since a significant (*p* ≤ 0.05) percentage of cell reduction in the G0/G1 phase with an increase of the G2/M phase percentage was detected, which was probably linked to the redox perturbation. Furthermore, an increase of early apoptotic and apoptotic/necrotic cells (mainly after 24 h of exposure to 12.5 and 3.0 μM BEA) was detected, alongside a loss of the mitochondrial membrane potential, particularly after a period of exposure of 72 h (from 2% to 95% with 1.5 μM BEA and from 10% to 80% with 3.0 μM BEA), which, indeed, may lead to cell death and apoptosis induction. DNA damage was also observed but only after exposure to high dosages (12.0 μM) [36].

Intestinal effects of BEA were also evaluated in terms of potential detrimental actions on the bacteria typical of the normal human intestinal flora [89], since the crucial role of the intestinal microbiota on health is well known [90,91]. It has been demonstrated that, indeed, BEA may have an impact on intestinal microbial balance, being able to inhibit, at dosages between 0.1 and 25 μg, several bacterial strains such as *B. pumilus*, *B. cereus*, *B. mycoides*, *B. sphaericus*, *P. alvei*, *P. azotofixans*, *P. macquariensis*, *P. pulvifaciens* and *P. validus*. BEA also inhibited anaerobes, namely *E. biforme*, *P. anaerobius*, *P. productus*, *B. adolescentis* and *C. perfringens*. Another work [81] studied the effect exerted by this mycotoxin on a panel of Gram-positive and Gram-negative bacteria, revealing antibacterial action against Gram-positive strains and *Mycobacterium* at concentrations ranging from 6 to 12.5 μM. Moreover, BEA can enter the bacterial membrane lipids and affect the integrity of the bacterial membrane structure, inducing permeabilization (at high dosages) and depolarization. BEA also inhibited the synthesis of bacterial macromolecules. On the other hand, Salim et al. [82] investigated the potential beneficial effects of *L. acidophilus* in the case of BEA exposure. They treated Caco-2 cells with 5 μM BEA and 2.5–5 × 105 CFU/mL of *L. acidophilus* (a popular probiotic) for 72 h, finding that at 12 and 24 h, the cell viability in the presence of *L. acidophilus* and BEA was higher (*p* < 0.05) than that obtained with BEA alone; however, at 48 and 72 h, it decreased. Nevertheless, this result revealed the interesting possibility of using probiotics to limit the detrimental health effects of mycotoxins. Another study evaluated [92] the nature of the interaction between BEA and 13 probiotic bacteria (*Bb. longum*, *Bb. bifidum*, *Bb. breve*, *Bb. adolescentes*, *Lb. rhamnosus*, *Lb. casei-casei*, *Lb. plantarum*, *E. crispatus*, *S. faecalis*, *S. termofilus*, *Lb. ruminis*, *Lb. casei* and *Lb. animalis*) typical of the gastrointestinal tract, carrying out fermentations in MRS broth added with 108 CFU/mL of the various strains and 5 mg/L BEA for up to 48 h. At the end of the fermentation period, the residual levels of BEA were analyzed, obtaining a reduction between 66.5% (*S. faecalis*) and 83.1% (*Lb. rhamnosus*), depending on the considered strain. Some mycotoxins were found adsorbed on the cell wall (2.5-8.7%) but mainly internalized in the bacterial cell. The quantity of internalized BEA augmented during the fermentation process (0.9 mg/kg at 4 h, 3.3 mg/kg at 48 h) and, at the end of the incubation period (*t* = 48 h), 67.5% (mean value) of the total amount of mycotoxin added at the beginning of the experiment was found inside the bacterial cells. Interestingly, BEA was able to interact with components of the bacterial wall: two adducts were identified via LC-MS, proving a possible mechanism involved in the loss of BEA toxicity [92].

In silico analysis is another useful tool for predicting the potential toxic effects exerted by emerging mycotoxins. Tran et al. [78] evaluated the physicochemical, pharmacological and toxicological properties of BEA by applying an in silico method (ACD/Percepta). This mycotoxin was classified as highly lipophilic, obtaining a logP value higher than the limit for bioavailability. BEA was also identified as a good substrate for Pgp, as it has been observed for other *Fusarium* mycotoxins and their metabolites [93], while intestinal peptide transporter 1 (PepT1) and intestinal bile acid transporter (ASBT) were not predicted to be implicated in BEA transport mechanisms. Altogether, these data support the validity of this method, opening a new possibility for toxicological modeling [78].

#### 2.1.2. Simulated Intestinal Environment

Research on BEA bioaccessibility has also been performed using a static and dynamic simulated gastrointestinal environment (Figure 1), mimicking salivary and gastrointestinal digestion as well as fermentation by human microbiota (Table 2). Meca et al. [94] evaluated the bioaccessibility of BEA (5–25 mg/L) in a model solution and wheat crispy bread added with different concentrations (1–5%) of natural binding compounds (dietary fiber, namely β-1,3 glucan, low-molecular-weight (LMW) chitosan, medium-molecular-weight (MMW) chitosan, fructooligosaccharides (FOS), galactomannan, inulin and pectin). BEA bioaccessibility obtained with the model solution was 31.8% after duodenal digestion and 54.0% after colonic fermentation, whereas the bioaccessibility in wheat crispy bread ranged between 1.9% (duodenal digestion) and 27.0% (samples that also underwent colonic fermentation). It follows that the prebiotic compounds employed were able to bind the mycotoxin just until colonic fermentation, which caused partial hydrolyzation of the dietary fibers, determining an increase in BEA bioaccessibility [94]. Another work investigated the bioaccessibility of BEA in wheat crispy bread using a static and dynamic simulated gastrointestinal environment [95]. The values obtained with the dynamic system (76.2–91.0%) were higher than those obtained with the static system (46.7–61.1%), probably because the first model performs a stronger agitation that likely breaks up the food matrix, determining an increase in BEA bioaccessibility. However, the addition of dietary fibers (inulin and FOS) was able to decrease BEA availability [95]. Other studies investigated the effects of dietary fibers and probiotic strains on BEA bioavailability [96,97]. Mallebrera et al. [96] found that probiotics (particularly *Lb. rhamnosus*) and prebiotics (mainly high-molecular-weight (HMW) cellulose) caused a significant reduction of BEA bioaccessibility (30–85% and 60–80%, respectively), while Ferrer et al. [97] obtained the best results in terms of BEA reduction with 5% cellulose (BEA bioavailability = 12.0% vs. 19.6% of the control) and *L. johnsonii* (BEA bioavailability = 6.6% vs. 11.3%). Taken together, these data corroborate the ability of lactic acid bacteria and dietary fibers to reduce the bioavailability of mycotoxins. Moreover, a BEA degradation product by colonic fermentation was identified [96].

### 2.2. In Vitro Effects of BEA on Species-Specific Intestinal Models

#### Cell Models

Species-specific toxicological effects of BEA have been investigated (Table 3) with the IPEC-J2 cell line (intestinal porcine epithelial cells) using traditional 2D in vitro cultures or two-compartment 3D culture systems with semipermeable Transwell^®^ inserts, obtaining cell polarization with the maturation of morphologically and functionally differentiated enterocytes [98] (Figure 1). Indeed, this model possesses tissue characteristics that are close to those of the porcine intestinal epithelium in vivo (i.e., expression of tight junction proteins, appropriate transport activities and TEER, etc.) and suitable for the study of porcine intestinal barrier functions [99]. Another species-specific intestinal cell line used for the toxicological evaluation of mycotoxins is IPEC-1, even if it is considered less morphologically and functionally differentiated in comparison with the IPEC-J2 cell line [100].

The cytotoxic effects of BEA (0–10 μM, 24 h of treatment) have been evaluated on proliferating and differentiated IPEC-J2 cells [50]. The exposure to this mycotoxin determined a decrease in IPEC-J2 viability. Comparing differentiated and proliferating IPEC-J2 cells, the latter were more susceptible to BEA cytotoxic effects. Interestingly, the IPEC-J2 cells seem more resistant to BEA with respect to Caco-2 cells. The treatment of proliferating IPEC-J2 cells with 5 μM BEA resulted in 15% early apoptotic and 3% late apoptotic/necrotic cells, while after 24 h exposure to 3 μM BEA, 34% of the Caco-2 cells were classified as early apoptotic and 31% as late apoptotic/necrotic [36]. BEA IC_50_ calculated on IPEC-J2 cells ranged from 2.24 (relative value) to 2.43 μM (absolute value) [101], while with the IPEC-1 cells, an IC_50_ value of 4.3 ± 1.8 μM (high toxicity) was obtained [102].

The effects of BEA (0–10 μM) alone and combined with DON (1.5–3 μM) on the intestinal barrier integrity were also evaluated [103]. At 5 μM, BEA significantly (*p* < 0.05) impaired the barrier function of the IPEC-J2 cells (action probably mediated through the induction of the phosphorylated mitogen-activated protein kinase (MAPK) ERK44/42) starting from 24 h of exposure and reaching an 80% TEER reduction after 72 h of exposure to 10 μM BEA, while cell viability was not affected at all the concentrations and combinations tested [103].

## 3. Enniatins

Enniatins (ENNs) are emerging mycotoxins synthesized by toxigenic microfungi mainly belonging to the *Fusarium spp*. [104] but also to other genera such as *Alternaria*, *Halosarpheia* and *Verticillium* [105]. These filamentous fungi are widespread pathogens of crops and represent a critical concern for food and feed safety worldwide [106,107]. Indeed, ENNs are commonly found on small cereal grains [107] and derived products in Europe [30,108], Africa [28,29], Asia [25,109], America [110] and Australia [111], with concentrations ranging from <1 μg/kg [25,29] to hundreds of mg/kg [28,108]. Other products can also be contaminated, such as dried fruits [34], nuts [34], eggs [35] and fish [112]. Chemically, ENNs are N-methylated cyclic hexadepsipeptides composed of three residues of D-2-hydroxyisovaleric acids alternated with three N-methyl-L-amino acids. To date, 29 ENN analogs have been isolated and characterized and, among them, ENNs A, A1, B and B1 are most frequently found in cereals [107]. ENNs possess several biological activities [104], including cytotoxic (several studies have shown that these mycotoxins can induce cell death in different cell lines [27,47,113,114,115,116,117,118,119,120,121,122,123,124,125,126]), antiviral [56], antibacterial [125,127,128], antifungal [129], insecticidal [59], anthelmintic [130], herbicidal/phytotoxic [131,132,133,134], anticancer [135] and immunotoxic [43] properties, which are thought to be related to the ionophoric properties of the ENN structure. Indeed, similarly to BEA, the presence of free electron pairs provides these molecules with peculiar electrophysiological characteristics that allow ENNs to co-ordinate with cations, forming weak chemical bonds primarily with metals like K^+^, Na^+^ and Ca^2+^ [136]. Once these lipophilic complexes have been formed, ions can be transported through cell membranes [2] and the result thereof is increased ion permeability that alters the intracellular ion concentrations inducing cell function disturbances [137]. ENN toxicity has also been associated with the ability of these compounds to set off mitochondrial dysfunctions (modifications of the mitochondrial membrane potential) [116,138], lysosomal alterations [113,117], cell cycle disruption [113,115,116,117,123] intracellular ROS production [113,116] (even if it should be reported that Dornetshuber et al. [38] found that ENN cytotoxicity is not linked to ROS generation and there are conflicting data on this aspect) and lipid peroxidation [116], although genotoxic effects have not been observed in vitro [104,116,118,126]. ENNs are also able to react with different protein substrates such as transporters and enzymes. For instance, it has been demonstrated that these mycotoxins bind not only to calmodulin-inhibiting phosphodiesterase [139] but also cholesterol acyltransferase (ACAT) functions [75] and interact with ATP-binding cassette (ABC) transporters ABCB1 (P-glycoprotein) [76,140], ABCC1 [140] and ABCG2 [76].

### 3.1. In Vitro Effects of ENNs on Human Intestinal Models

#### 3.1.1. Cell Models

Studies have been conducted on ENNs to evaluate their potential toxic effects using intestinal cell lines (i.e., Caco-2 cells, HT-29 cells, etc.) in traditional culture systems as well as in two-compartment transwell models, but also applying newly available in silico methods [78] (Table 4).

Meca et al. [141] used duodenal fluid from the simulated gastrointestinal digestion of 3 g of wheat crispy bread spiked with ENN A, A1, B and B1 at 1.5 and 3.0 μmol/g to evaluate the intestinal absorption (transport profile) and bioavailability of these mycotoxins using a two-compartment Caco-2 cell system (Figure 1). The incubation medium containing the digestion fluid was added to the luminal (Ap) side of the model and aliquots of the medium were collected from the Bl compartment after 1, 2, 3 and 4 h of exposure. ENN A1 (3.0 μmol/g) was the compound that demonstrated the highest transport percentage (73.8 ± 0.9% at 4 h). On the contrary, ENN A (3.0 μmol/g) showed the lowest transportability (50.7 ± 1.3% at 4 h). As for the ENN bioavailabilities (calculated through the determination of the ENN concentrations in the Bl compartment at the end of the incubation time), the highest (60.0 ± 1.0%) and lowest (40.8 ± 1.0%) values were found for ENN A and B, respectively. However, generally, the calculated ENN bioavailability percentages were often below or around 50%; thus, half of the amount of the mycotoxin digested was not transported across the intestinal barrier and was unable to exert its potential toxicological effect on target organs/tissues [141]. Other in vitro models have been developed using Caco-2 cells alone or cultured with bacterial strains typical of the intestine (*L. animalis*, *Lb. casei*, *Lb. casei rhamnosus*, *Lb. plantarum*, *Lb. rhuminis*, *Lb. casei-casei*, *Bifidobacterium breve*, *Bif. adolescents* and *Bif. bifidum*) to mimic the duodenal and colonic intestinal tract, respectively [142], in order to assess ENN bioavailability (ENN A, A1, B and B1). The obtained transepithelial transport profiles highlighted a progressive passage of the mycotoxins from the Ap to the Bl compartment depending on the incubation time, molecular structures and concentrations [143] of the ENN involved. As for the duodenal bioavailability (calculated as the sum of the absorption values at the end of the incubation time), the highest value was obtained for the 1.5 μM ENN A (197.1 ± 4.2% of bioavailability), while 3.0 μM ENN A and B showed the lowest bioavailability percentages (148.5 ± 4.2% and 151.3 ± 4.6%, respectively), results that were in line with those obtained from another research of the same author [141]. Concerning the colonic transport, the highest number of ENNs was found in the Bl side, followed by the cell matrix and the Ap side (where the number of ENNs was nearly half of that found in the Bl compartment). As for the latter, mycotoxin concentrations were similar between all the ENNs. Furthermore, the highest bioavailability was for that of ENN B1, followed ENN B, A1 and A. Tran et al. [78] studied ENN (ENN A, A1, B and B1) bioavailability (in coexposure with silibinin) using a two-compartment transwell model of Caco-2 cells cocultured with RAW 264.7 cells (murine macrophages). ENN A and A1 were found in higher concentrations in the cells and ENN B and B1 in the basolateral medium. Since the total number of ENN A, A1, B and B1 recovered was significantly lower than the quantity added at the beginning of the test, metabolization had been hypothesized and the nontarget U-HPLC-MS analysis detected the presence of an ENN B metabolite (M6). ENNs were also found in the cells (in RAW 264.7 as well as in Caco-2 cells), probably because these compounds, due to their high lipophilicity, are able to interact with the cellular membrane, as indicated by the in silico prediction of ENN physicochemical, pharmacological and toxicological properties [78]. Moreover, the possible involvement of transporters in the intestinal absorption of ENNs has been evaluated on Caco-2 cells [140]. The absorption profile showed that the permeability of ENN B1 in the Bl to Ap direction was 6.7-times higher than in the opposite direction. No significant effects have been observed after treatment with a BCRP inhibitor (fumitremorgin C) in the Ap to Bl transport of ENN B1. However, it increased significantly after treatment with Pgp (verapamil) and MRP2 (MK571) inhibitors, revealing that the Ap transporters MRP2 and Pgp may be involved in the ENN B1 intestinal transport [140]. Finally, in a recent study, Tran et al. [78] performed an in silico prediction (using ACD/Percepta) of the physicochemical, pharmacological and toxicological properties of ENNs, obtaining a very low aqueous solubility with a resulting low bioavailability for these compounds. Moreover, ENNs were predicted as weak substrates for Pgp (weaker than BEA), and PepT1 and ASBT were not found connected to ENN transport [78].

ENNs are able to exert cytotoxicity on intestinal cells (i.e., Caco-2, HT-29) at low micromolar concentrations, showing a cytotoxic effect on Caco-2 cells that can be three- to four-fold higher than BEA [80], although no effects have been observed on Caco-2 cells up to concentrations of 50 nM ENNs [78]. Dornetshuber et al. [123] evaluated ENN cytotoxicity on Caco-2 cells, obtaining an IC_50_ of 1.99 ± 0.09 after 72 h of exposure. Moreover, exposing HCT116 cells with homozygously disrupted p53, p21 or bax genes to ENN (0–10 μM, 24 h), a p53-independent cytotoxic activity was observed [123]. As for the toxicity exerted by the different ENNs, Meca et al. [144] evaluated the effects of ENN A, A1, B, B1 on Caco-2 cells, finding that only ENN A1 and B1 affected cell viability, while ENN A and B did not cause any cytotoxic effect at the tested concentrations (0–30 μM, 24 h) [144]. In particular, the IC_50_ (24 h) values were estimated only for ENN A1 (12.3 μM) and B1 (19.5 μM), while no IC_50_ were obtained for ENN A and B with the applied concentrations (0–30 μM, 24 h) [141]. On the other hand, in a comparative cytotoxicity study on ENNs performed using Caco-2 and HT-29 intestinal cells, intense cytotoxic effects have been observed after the exposure to ENN A, A1, B1 and B4, with ENN A1 being the most toxic compound in both the cell lines, showing IC_50_ values at 48 h of 2.7 ± 0.8 μM for Caco-2 cells and 1.4 ± 0.7 μM for the HT-29 cells [120]. On the contrary, ENN J3 did not exert any cytotoxic effects at all the concentration tested, neither on differentiated nor in undifferentiated Caco-2 cells [145]. Another study evaluated ENN cytotoxicity on human gastric (N87) and Caco-2 cells [81], finding a higher sensitivity to these mycotoxins in comparison to Caco-2 for the first cell line with an IC_50_ range between 0.003 ± 0.002 μM (ENN A1) and 1.7 ± 0.1 μM (ENN B), while the Caco-2 cells showed an IC_50_ range between 1.1 ± 0.2 μM (ENN A) and 4.6 ± 1.3 μM (ENN B) [81]. Since co-occurrence is a frequent event in the case of mycotoxin contamination of food and feedstuffs, the interaction effects of ENNs (ENN A, A1, B and B1) have also been investigated. The results obtained using a Caco-2 cell model indicated that binary, tertiary and quaternary coexposures may produce mostly additive effects on cell viability (only the coexposure ENN B + B1 produced antagonism) event if synergistic effects (i.e., ENN B + A1, ENN B1 + A1 and ENN A + A1 + B) have also been observed [122]. Another work [146] evaluated the cytotoxic actions of binary mixtures of ENN B and other *Fusarium* (aurofusarin, deoxynivalenol, nivalenol and zearalenone) and *Alternaria* (tenuazonic acid) mycotoxins, mimicking the typical mixtures occurring in natural contaminations of cereal grains. For all the combinations tested, synergistic effects were not observed, while binary mixtures (at cytotoxic concentrations) of ENN B with deoxynivalenol, nivalenol, zearalenone and tenuazonic acid produced antagonistic effects [146]. The interaction effects of mixtures of ENN B, deoxynivalenol (DON) and alternariol (AOH) on Caco-2 cells have also been evaluated, finding that the higher concentrations of ENN B and DON in coexposure (48 and 72 h) exerted higher cytotoxic effects than DON alone. However, it should be noted that binary and even tertiary associations of DON, ENN B and AOH resulted in interactions that had no uniform patterns and synergistic, additive or antagonistic effects have been observed depending on the concentrations and time of incubation applied. Nevertheless, as the presence of mycotoxin combinations may enhance the toxicological effects, co-occurrence is an issue that needs to be investigated further [147].

Mechanisms of ENN toxicity have been investigated using in vitro intestinal models. Ivanova et al. [113] evaluated ENN B toxicity mechanisms on Caco-2 cells. Significant cytotoxicity was observed in connection with the lysosomal functionality (lysosomal membrane permeabilization was noticed with the release of cathepsins into the cytosol). Cell cycle arrest was also detected, with an increase of cells in the G2/M phase, leading to the beginning of cell death cascades. Indeed, a dose-dependent increase in necrotic cells with a corresponding decrease in the number of normal cells was recorded. On the contrary, the population of apoptotic cells reached a maximum percentage of 2% after exposure to 1 μM ENN B, showing a decrease at higher concentrations. Thus, ENN-B-induced cell death follows a necrotic, rather than an apoptotic, path and the lysosomal damage appears as an early event in ENN B toxicity. Mitochondrial membrane permeabilization and an increase in ROS production were also observed; however, antioxidant treatments did not affect the lysosomal damage, suggesting that ROS production is not the initial stimulus of the toxicological effects induced by ENN B [113]. Another work [116] evaluated the various toxicity mechanisms of different types of ENNs (ENN A, A1, B and B1) using Caco-2 cells. ENN A and A1 showed lower IC_50_ values through MTT as well as NR assays, with the latter being the most sensitive. ENN toxicological effects involved early ROS production, which was concentration- and time-dependent and already present at the initial stage of the exposures (for ENN A1, B and B1), and increased MDA production (which may lead to further cell damage, in particular via lipid peroxidation (LPO) induction). The mitochondrial membrane potential showed a dose- and time-dependent decrease, and cell cycle disruption was also demonstrated with alterations (time- and concentration-dependent) in the percentages of cells in the SubG0/G1, G0/G1 and G2/M phases for all the ENNs tested. Furthermore, ENN A, A1 and B1 were able to arrest the cell cycle at G2/M after 24 h of exposure, and after 72 h of treatment, an arrest in the S phase was observed mainly for all the evaluated compounds. ENNs also induced apoptotic and necrotic effects via the mitochondrial pathway [116]. Apoptosis was mainly observed after 24 and 48 h of treatment, while necrotic effects were detected for all the ENNs after 72 h. ENN A, A1 and B1 also determined significant DNA damage but not ENN B. This latter ENN deserves specific mention because it is less cytotoxic than the other ENNs, probably because of its lipophilicity rate (ENN A > ENNA1 > ENNB1 > ENNB). After 24 h of ENN B exposure, no significant effects were found in terms of cell cycle disruption, apoptosis and/or necrosis induction, DNA damage and mitochondrial membrane potential perturbation. Nevertheless, ENN B (3 μM) caused ROS generation and LPO; thus, for this mycotoxin, ROS and LPO production are not responsible for apoptosis induction [116]. ENN cytostatic action on intestinal cells was also observed by Dornetshuber et al. [123], who exposed HCT116 cells with homozygously disrupted p53, p21 or bax genes to ENN (0–10 μM, 24 h) and found a p53-dependent cell cycle arrest in the G0/G1 phase.

ENNs are also known for their antibacterial action [104]. This property has been evaluated in an intestinal in vitro environment. The antimicrobial activity of ENN B against some pathogens of the gastrointestinal tracts (*E. coli*, *E. faecium*, *S. enterica*, *S. dysenteriae*, *L. monocytogenes*, *Y. enterocolitica*, *C. perfringens*, *P. aeruginosa* and two strains of *S. aureus*) has been investigated and, contextually, the cytotoxic effects of mycotoxin on differentiated and undifferentiated Caco-2 cells were assessed [125]. ENN B was able to inhibit the growth of several strains of micro-organisms, which are considered normal pathogens of the intestinal tract, with the most potent effects observed on *C. perfringens* and *S. aureus* CECT 976. No activities have been displayed on *S. aureus* CECT 240, *E. coli* CECT 4782 and *S. dysenteriae* CECT 584. Decreased viability of the Caco-2 cells was detected after exposure to concentrations ≥6 μM for ENN B (both undifferentiated and differentiated cells) but the IC_50_ value was not reached in the range of concentrations and time of exposure applied to obtain the antibacterial action (neither for the undifferentiated nor for the differentiated cells). This proves that this mycotoxin has the ability to inhibit the growth of some gastrointestinal pathogens at nontoxic concentrations for differentiated and undifferentiated intestinal cells (Caco-2 line) [125]. Another study [81] evaluated the effects of different ENN analogs on a panel of Gram-positive and Gram-negative bacteria. ENNs showed antibacterial activities against Gram-positive strains and *Mycobacterium* (ENN A appeared as the most active. ENN B was less active with MIC > 100 μM for all the evaluated Gram-positive bacteria with the exception of *C. perfringens*) at concentrations ranging from 3.12 to 100 μM, which was able to alter the integrity of the bacterial membrane by embedding into the structural lipids causing permeabilization (at high concentrations of exposure) and depolarization and determining the inhibition of the synthesis of bacterial macromolecules (at lower doses). The effects of ENNs (A, A1, A2, B, B1 and B4) on probiotic strains (lactic acid bacteria like *Bf. longum*, *Bf. bifidum*, *Bf. breve*, *Bf. adolescentis*, *Lb. rhamnosus*, *Lb. casei-casei*, *Lb. plantarum*, *Lb. paracasei*, *Lb. ruminis*, *S. thermophilus*, twenty-two strains of *S. cerevisiae* and nine strains of *B. subtilis*) commonly present in the human intestine have also been evaluated to understand the potential detrimental actions of naturally occurring emerging mycotoxins on the microbiota [127]. The results showed that ENNs exerted inhibitory effects against many of the tested bacteria, with ENN A1 being the most active (reduction of the growth of eight strains at a dose of 20,000 ng), followed by ENN B1 (inhibition of six strains). On the contrary, ENN B and B4 did not display any antimicrobial action at the tested concentrations (up to 20,000 ng). Thus, ENNs have the ability to perturb the equilibrium of the microbiota, with potential significant repercussions on health.

#### 3.1.2. Simulated Intestinal Environment

Many studies concerning ENN bioaccessibility and their catabolic fate have been carried out using in vitro simulated gastrointestinal models (Table 5, Figure 1). For instance, Meca et al. [141] used a simulated gastrointestinal environment to evaluate the bioaccessibility of ENN A, A1, B and B1 into gastric and duodenal fluid after the in vitro digestion of 3 g of 1.5 and 3.0 μmol/g spiked commercial wheat crispy bread. ENN B (at 1.5 μmol/g, 68.6 ± 2.9%) and A1 (1.5 and 3.0 μmol/g, 72.6 ± 1.8% and 70.0 ± 1.7%, respectively) were the two mycotoxins that showed the lowest duodenal bioaccessibility rate. On the other hand, ENN A (at 3 μmol/g, 87.3 ± 2.9%) was the compound that showed the highest bioaccessibility rate [141]. Generally, considering all the concentrations tested, a reduction of 9 to 30% in the amount of mycotoxins initially present in the crispy bread has been observed [141]. Further, the effects, in terms of ENN bioavailability, of the addition of prebiotic ingredients (1, 5 and 10% inulin) into the crispy bread (similarly spiked with 1.5 and 3.0 μM ENN A, A1, B and B1) have been investigated [148]. The best results in terms of bioaccessibility reduction were obtained with 5 and 10% inulin, which provided percentages between 58% (3.0 μM ENN A1) and 74% (1.5 μM ENN B1) and between 51% (3 μM ENN A) and 74% (1.5 μM ENN B1), respectively. It is likely that the inulin in the gastrointestinal fluid may arrange in reticular structures able to absorb part of the ENNs. This capability seems to be directly related to the quantity of fiber added [148]. In addition, Manzini et al. [95] evaluated the effects of inulin and fructooligosaccharides (FOS) on ENN bioaccessibility in wheat crispy bread, obtaining ENN bioavailabilities of 32.7 and 23.0% in the inulin-enriched products (1 and 5% *w*/*w*, respectively), values that were 29.0–50.5% lower than the control. Similar results have been obtained with FOS, demonstrating that these prebiotic compounds are able to decrease the risk related to the ingestion of emerging mycotoxins in grain-based products [95]. Another work [97] evaluated the influence of prebiotic compounds (cellulose and inulin) as well as food ingredients (milk whey, β-lactoglobulin and calcium caseinate) and probiotic micro-organisms on ENN (Ens A, A1, B, B1) bioaccessibility in wheat crispy bread. The best results in terms of ENN reduction were obtained with the addition of 5% sodium caseinate (ENN gastric and duodenal bioavailability = 20.3 and 17.0% vs. 39.6 and 33.4% of the control) and L. johnsonii (ENN gastric and duodenal bioavailability = 27.8 and 21.2% vs. 39.6 and 33.4% of the control). Thus, different components (food ingredients, probiotics and prebiotics) may significantly reduce mycotoxin bioaccessibility binding or degrade these natural contaminants. This evidence needs to be considered, since the addition of these beneficial compounds may represent an advantageous and low-cost method to diminish the risk related to mycotoxin intake with food and feed [97]. A simulated gastrointestinal environment was also applied to assess the bioaccessibility of ENNs (ENN A, A1, B and B1) added in two concentrations (1.5 and 3.0 μmol/g) to different types of commercial breakfast cereals, cookies and breads [149]. In general, the highest ENN bioavailability percentages were found in white loaf bread (1.5 μM ENN A = 79.9 ± 2.8%; 1.5 μM ENN A1 = 64.2 ± 2.4%; 1.5 μM ENN B = 69.8 ± 2.9%; 1.5 μM ENN B1 = 73.6 ± 2.2%), whereas the lowest values were detected in wheat bran with fibers (1.5 μM ENN A = 50.1 ± 3.1%; 1.5 μM ENN A1 = 40.4 ± 1.9%; 1.5 μM ENN B = 43.9 ± 3.4%; 1.5 μM ENN B1 = 46.3 ± 3.1%). Given these results, the food matrix (composition, structure, presence of fibers able to adsorb active compounds) seems to be a strong influencing factor in ENN bioaccessibility and, in particular, the presence of fibers. Indeed, several studies have pointed out that the presence of certain amounts and types of fibers can reduce the percentage of mycotoxin bioavailability as natural adsorbing materials [5,150]. ENN bioavailability was also assessed in a follow-up infant formula [151]. None of the analyzed samples (*n* = 72) were contaminated with ENN B; thus, the bioavailability of this mycotoxin has not been evaluated. Only one sample was found positive for ENN A, with a colonic + duodenal bioaccessibility of 1.63 ± 0.01%, while ENN A1 and B1 showed a bioaccessibility range of 4.36 ± 0.61%–60.53 ± 2.64% and 1.49 ± 0.04%–8.43 ± 0.64%, respectively. Even if higher values were found for the colonic bioaccessibility in comparison with the duodenal one, just a fraction of the ENNs found in the samples was bioavailable, suggesting that the macronutrients in these formulations, like the fibers in the aforementioned products, may bind the mycotoxins and reduce their activity [151]. An in vitro gastrointestinal model (simulated digestion and colonic fermentation by typical human intestinal microbiota) has also been applied for the investigation of the catabolic fate of ENN B [105]. Significant degradation of the parent compound has been observed (78.45 ± 5.39%), and additional degradation of the residual mycotoxin has been detected after 24 h of colonic fermentation. Five catabolic metabolites (oxidation and N- demethylation products) characterized by higher hydrophilicity than the parent compound have been isolated (M1–M5). Moreover, the pharmacokinetic characteristics of these metabolites were evaluated in silico with BOILED-Egg predictive model, finding that two degradation products (M4 and M5) were probably not absorbed at a gastrointestinal level, while M2 and ENN B may be absorbed. M1 and M3 showed possible moderate crossing of the blood–brain barrier where no interaction with Pgp was predicted and M1, M2, M4 and M5 were classified as likely CYP2C19 inhibitors. These data suggest that toxicological evaluations of mycotoxins, besides assessing the hepatic metabolism of these compounds, must not ignore the degradative biotransformation taking place into the digestive tract, which is intense and crucial to perform a solid toxicological risk assessment. Thus, given these results, the application of simulated gastrointestinal systems seems to be a reliable and convenient in vitro method to evaluate the catabolic fate of mycotoxins into the gastrointestinal tract as well as the oral ENN bioaccessibility in cereal and cereal products since indications of a close correlation between in vitro bioaccessibility and in vivo bioavailability have been provided for several mycotoxins [152].

### 3.2. In Vitro Effects of ENNs on Species-Specific Intestinal Models

#### 3.2.1. Cell Models

The species-specific intestinal toxicological effects of ENNs have been investigated mainly with porcine and bovine cell lines (Table 6). Kolf-Clauw and colleagues [153] evaluated the intestinal toxicity of ENN alone and in coexposure with T-2 toxin (T2) on IPEC-1 cells using cell proliferation as an endpoint for cytotoxicity. A dose-dependent decrease in IPEC-1 cell proliferation was observed after the exposure to ENN and T2 alone and in association. In particular, ENN caused a decline in cell proliferation at all the concentrations applied (0.3–100 μM, for 48 h; 96.2–13.6% proliferation in comparison with the control). The exposure to the mixture T2 + ENN at a 1:1000 ratio determined, again, a dose-dependent action on cell proliferation but less than additive effects were observed at IC_50_ values. Increasing antagonism was detected with decreasing dosages of mycotoxins. Similar results have been obtained using a swine jejunal explant (ex vivo exposure); indeed, comparing the data obtained with these two swine intestinal models, a good correlation was obtained (*r* = 0.98). These findings reinforce the reliability of species-specific intestinal models and, in addition, point out the need to further consider the effects of mycotoxin coexposures, which are common in the case of the ingestion of naturally contaminated products. [153]. IPEC-1 cells were also used by Khoshal et al. [102] to assess the cytotoxicity of individual ENNs (A1, B and B1). ENN A1 (IC_50_ ENN = 1.6 ± 0.3 μM) were more toxic than ENN B (IC_50_ ENN B = 4.4 ± 0.9 μM), while ENN B1 (IC_50_ ENN B1 = 13.5 ± 2.5 μM) was the least toxic. All the tested mycotoxins showed dose-dependent cytotoxicity toward the intestinal epithelial cells. Moreover, the combined effects of individual ENNs associated with DON were evaluated. The cytotoxicity of ENN A1, B and ENN B1 in coexposure with DON was similar to or lower than the toxicity of DON alone at all the concentrations applied, demonstrating that the co-occurrence of ENNs and DON does not increase the toxic effect of this cell line [102]. ENN cytotoxicity (ENN A, A1, B and B1; 0–100 μM, 24 h of treatment) was also evaluated in relation to the differentiation status of the IPEC-J2 cells [50], finding no significant differences between proliferating and differentiated cells in terms of ENN sensitivity. ENN A was the most cytotoxic, followed by ENN A1, B1 and, lastly, ENN B. Slightly different results have been obtained by Novak et al. [101] on the same intestinal cell model (IPEC-J2 cells), who found the lowest IC_50_ value for ENN B (3.25 μM), followed by ENN A (IC_50_ = 3.40 μM), ENN B1 (IC_50_ = 3.67 μM) and ENN A1 (IC_50_ = 4.15 μM). IPEC-J2 cells seem to be less sensitive to ENNs in comparison with Caco-2 cells. Indeed, after 24 h of exposure of proliferating IPEC-J2 cells to a 5 μM concentration of the most cytotoxic ENN of this study (ENN A) and ENN A1, low percentages of 4.1%, 3.5% early apoptotic and 2.7% and 2.4% late apoptotic/necrotic cells, respectively, were detected [50]. On the contrary, after the exposure of Caco-2 cells to 3 μM ENN A and A1, remarkably higher percentages of early apoptotic (25% and 24%, respectively) and late apoptotic/necrotic (34% and 27%) cells were observed [116]. The potential detrimental effects of the intestinal barrier integrity of ENNs (0–10 μM) alone and combined, in the presence or absence of DON (0–3 μM), were also tested using the IPEC-J2 cell model [103]. ENN B showed the most intense effects in terms of TEER reduction (barrier impairment), followed by ENN B1, A and A1. The association of ENNs, which individually had no activity on TEER, seemed to produce an additive effect, while the addition of DON to the ENN mixtures did not intensify the ENN-induced TEER reduction. It should be noted that the observed TEER decreases were not determined by cytotoxic effects, since the viability of the cells was affected neither by the mycotoxin alone nor by the mixtures tested [103]. Another species-specific intestinal cell model used to evaluate ENN toxicity comprises calf small intestinal epithelial cells B [154]. Reisinger et al. applied this in vitro system for the assessment of ENN B cytotoxicity, using different assays for studying metabolic activity (WST-1 assay), lysosomal activity (NR assay) and total protein content (SRB assay) of this cell line. IC_50_ values of 6.7 and 4.0 μM were found with the WST-1 and NR assay, respectively, while the IC_50_ value for the SRB assay was impossible to obtain since the protein content of the cells did not drop below 50%. Given these results, it seems that the bovine intestinal cells are as sensitive to ENN B as the human Caco-2 cells and the swine intestinal cell models (IPEC-1 and IPEC-J2 cells). Thus, even if the bovine species is regarded as less affected by the adverse toxicological effects of mycotoxins [155], particularly due to the degradations operated by the ruminal flora, the ingestion of mycotoxins resistant to ruminal degradation, alterations of the ruminal flora or the possibility that a certain proportion of mycotoxins might bypass the rumen may cause the exposure of the bovine intestinal epithelium to these compounds. Detrimental effects on animal health and production cannot be excluded [154].

#### 3.2.2. In Vitro Rumen Models

Evidence indicating mycotoxin-associated health issues has also been reported in ruminants [156,157], animals that have commonly been regarded as less susceptible to mycotoxin toxicity [158]. Debevere et al. [159] developed an in vitro rumen model including a ruminal inoculum and feed matrix to investigate the fate of several mycotoxins (a mixture of 60 mg/kg nivalenol, 12 mg/kg DON, 1 mg/kg ENN B, 6 mg/kg mycophenolic acid, 2 mg/kg roquefortine-C and 3 mg/kg zearalenone spiked in maize silage, to reach concentrations to mimic a realistic contamination) subjected to ruminal digestion for up to 48 h. At the beginning of the incubation period, an increase in the relative ENN B concentrations was observed, probably due to the release of adsorbed mycotoxin, followed by a decrease. Moreover, when the pH was maintained in a physiological range (around 6.8), the relative concentration of ENN B decreased more rapidly and a greater amount of mycotoxin was degraded (72%, 48 h of incubation), while at a lower pH condition (as in case of subacute ruminal acidosis), ENN B degradation was hindered. Another influencing factor was the type of rumen inoculum. ENN B degradation was more efficient with the inoculum of lactating cows rather than with that of nonlactating animals (which can frequently have rumen acidosis and lower ruminal microbial activity). Thus, depending on the ruminal conditions (low pH or microbial activity), part of ENN B can pass through the rumen, reaching the intestine unmodified [159]. As a consequence, mycotoxin control gains new importance in these species for the benefit of ruminant health and production. An in vitro rumen model was also applied to screen the efficacy of different commercial mycotoxin detoxifier agents [160]. In particular, with regard to ENN B, three binding products were tested (Binder 1: clay minerals and yeast derivatives; Binder 2: bentonite, leonardite, plant extracts, epoxidase; Binder 3: bentonite and sepiolite). The results demonstrated that all the binders partly adsorbed ENN B; however, while Binder 1 stably adsorbed the mycotoxin by 24% during all the incubation period, Binders 2 (28%) and 3 (22%) showed a reversible bond (desorption) since the differences between the ENN B concentration in the control and treated groups decreased over time [160]. Nevertheless, in vitro rumen models (Table 7) proved to be a suitable and flexible tool to investigate mycotoxin bioavailability in these species.

## 4. Occurrence of Beauvericin and Enniatins in the Food Chain Products

The EFSA scientific opinion concluded that BEA and ENNs are substantially stable during commercial cereal processing, including hot-drying and ensiling procedures, and acute exposure to these compounds does not indicate concern for human health. The EFSA also underlined the lack of human toxicity data concerning chronic exposures and LOAELs/NOAELs for livestock species [16]. Nowadays, several studies have begun paying more attention to investigate the occurrence of BEA and ENNs in food, feed and animal food derivative products and the relative risk posed to human and animal health. A nationwide survey on rice contamination in Brazil showed a high occurrence of BEA (>70%, with a mean concentration of 23.33 μg/kg in flour samples) and, among ENNs, just one and two samples (*n* = 93) were found positive for ENN B (mean concentration < LOQ) and B1 (mean concentration 1.45 μg/kg), respectively [161]. In wheat samples cultivated in Romania, a higher occurrence of emerging mycotoxins was found in the harvest year 2014 (69% of the wheat samples) in comparison with 2015 (40% of the wheat samples). The highest maximum concentrations were measured for ENN B (814.6 μg/kg) in the harvest year 2015 [162]. In Belgian wheat samples (2015–2016 growing season), the incidence of ENN A, A1, B, B1 and BEA was high (64%, 73%,100%, 100% and 100% of the analyzed wheat samples, respectively). The maximum contamination levels were measured for ENN A1 (477.5 μg/kg), ENN B (2168 μg/kg) and ENN B1 (776.7 μg/kg) [163].

The transfer of these mycotoxins from feed to animal food derivative products has already been reported. In recent papers, Nácher-Mestre et al. [164] showed that ENN B and BEA present in fish feed at average concentrations of 19.9 μg/kg and 30 μg/kg were not transferred in fish samples (quantification limit: 0.1 μg/kg), while Tolosa et al. [112] found them in edible tissue from farmed fish. The occurrence of ENN B, B1 and A1 was detected in 40%, 33% and 25% of the tested fish samples, respectively [112]. The effects of contaminated feed in the broiler chickens’ diet were also assessed [165]. ENNs (1.6–2.7 mg/kg) and BEA (0.03–0.5 mg/kg) were detected in feed samples. A strong positive correlation between feed conversion ratio (FCR) of birds and the exposure to ENNs (*R*^2^ = 0.60) and BEA (*R*^2^ = 0.73) was found, indicating that these mycotoxins may have a negative effect on birds’ performances. Another interesting research was conducted on pooled breast milk samples to assess possible contaminations by BEA and ENNs. The highest concentration of 6.2 ng/L was found for BEA, while ENN B was present at a level of 4.7 ng/L and ENN B1 was detected below its respective sample LOQ value [166].

Table 8 reports the results of different studies on the presence of BEA and ENNs in food and feed. These mycotoxins were found in most of the samples in a very variable concentration, probably because of the different analytical methods. Since these compounds are frequent contaminants of food and feedstuffs, it is important to include them in food chain product evaluations. Furthermore, the assessment of potential interactions between BEA and ENNs and other associating mycotoxins should be performed together with the investigation of possible chronic exposures.

## 5. Legislation

Regulatory limits for mycotoxins or at least guidance levels have been established in many countries, to ensure food and feed safety reducing implications for public health. In the European Union, the European Commission has defined strict rules or guidance levels for a number of toxins in different matrices.

According to the general provisions, set in the consolidated version of Council Regulation (EEC) 315/93 concerning procedures for contaminants in food, contaminant levels should be kept as low as reasonably achievable (ALARA) following the recommended good working practices. When necessary for protecting public health, maximum levels should be established for specific contaminants as well as procedures for setting such levels [173]. The European legislation stipulates maximum admissible levels for mycotoxins in foodstuff in the valid version of the Commission Regulation (EC) 1881/2006 that has been updated regularly to meet new scientific findings [174]. So far, specific mycotoxin regulations only cover some of the known mycotoxins according to the availability of toxicological characterization and occurrence data, which, in turn, depend on the existence of methods of sampling and analysis [175,176]. The regulations being enforced encompass 13 different mycotoxins or groups of mycotoxins classified as relevant for food and feed safety: aflatoxins B1, B2, G1, G2 and M1; fumonisins B1, B2 and B3; ochratoxin A, deoxynivalenol, zearalenone, HT-2 toxin and T-2 toxin. The emerging mycotoxins are not covered by legislation. This is the case of modified mycotoxins (often called “masked” mycotoxins, as their chemical structure is changed by the host plant of the fungus or by the fungus itself) as well as other emerging mycotoxins (such as ENNs and BEA) that are insufficiently toxicologically characterized and for which a significant amount of knowledge is still not available. Missing regulations drive gaps in routine mycotoxin analysis in the European Union; therefore, many of these compounds are neither routinely determined nor monitored and can go undetected. As a result, they represent a threat to both human and animal health, while the overall levels of contamination figure being below legislative limits [176,177]. Maximum levels for mycotoxins not covered by legislation are under discussion. Meanwhile, more studies are needed to reveal the toxicity of these compounds and the possible interaction between the different mycotoxins present in each matrix. Furthermore, new reliable analytical methods should be developed for the determination of emerging mycotoxins. In fact, proper sampling procedures play a crucial part in the determination of the contamination levels. The Commission Regulation (EC) 401/2006, which reports the methods of sampling and analysis for the official control of the levels of mycotoxins in foodstuffs (consolidated version) [178], only covers aflatoxin B1, aflatoxin M1, total aflatoxins, ochratoxin A, patulin, *Fusarium*-toxins, zearalenone and citrinin. Regarding the sampling methods and analysis of mycotoxins in feedstuffs, the Commission Regulation (EC) 152/2009 sets provisions for the detection of aflatoxins [179]. Once again, regulations have to evolve in this regard. In order to minimize the contamination of foodstuffs and feedstuffs, over the last years, alongside the existing rules, several commission recommendations have been adopted in relation to the presence of mycotoxins, in particular ochratoxin A, deoxynivalenol, zearalenone, fumonisins, T-2 and HT-2 toxin in cereals and cereal products intended for animal feed or both for food and feed. In addition, the influence of European organizations and programs on the EU mycotoxin regulatory developments is significant (e.g., European Food Safety Authority Opinions; Rapid Alert System for Food and Feed, involving rapid information exchange about food or feed safety between the competent authorities of the member states, in the event of risks in the food chain; European Union Reference Laboratory mycotoxins and plant toxins in food and feed). On these bases, it can be concluded that the growing awareness of the risks posed by mycotoxins is supporting regulatory developments that will ensure the improvement and harmonization of more comprehensive legal limits for mycotoxins in the European Union in order to address emerging challenges and to better guarantee safe and healthy foodstuffs and feedstuffs.

## 6. Conclusions and Future Directions

Food and feedstuff contamination by emerging mycotoxins is a global issue, since high occurrences and sometimes even remarkably high concentrations have been observed in several geographical regions. To date, numerous in vitro and in vivo studies have pointed out the potential adverse effects of these natural contaminants, highlighting the fact that they represent a serious threat to human, animal and environmental health. The present lack of data on mycotoxin interaction, toxicity and toxicokinetics has so far prevented a comprehensive risk assessment on these mycotoxins and, consequently, to establish regulatory limits. Thus, more studies are urgently needed to fill the knowledge gap. Particularly, research on the intestinal effects of the emerging mycotoxins is required, since the gastrointestinal tract is a crucial site, which, besides being a potential target organ, influences mycotoxin bioavailability and metabolism. The interaction between mycotoxins, microbiota and mucus is worthy of further consideration in light of recent research that has pointed out the crucial interplay occurring between these elements in determining mycotoxin toxicity.

## Figures and Tables

**Table 1:** In vitro effects of and beauvericin (BEA), on human intestinal cell models.

Models/Cells	Concentration and Exposure Time	Method/Assay	Effects	Ref.
ACD/Percepta (in silico model)	BEA	In silico toxicity analysis	High lipophilia and low bioavailability (log *p*-value higher than the limit for bioavailability) No genotoxicity Caco-2 permeability (Pe) = 5.8 10^−4^ cm/s Good substrate for Pgp (*p* = 0.9, RI = 0.38)	[78]
Caco-2 cells	0–10 μM BEA; 24 and 48 h	HCA multiparameter assay	Cytotoxicity ((BEA) ≥ 1 μM)	[37]
Caco-2 cells	0–100 μM BEA; 48 h	Resazurin-based in vitro toxicity assay	Cytotoxicity IC_50_ = 3.9 ± 0.7 μM)	[81]
Caco-2 cells	0–30 μM BEA; 24 and 48 h	MTT assay	Cytotoxicity (IC_50_ at 24 h = 20.62 ± 6.9 μM; IC_50_ at 48 h = 12.75 ± 4.8 μM)	[79]
Caco-2 cells	0–50 nM BEA; 72 h	Resazurin-based in vitro toxicity assay	No cytotoxicity	[78]
Caco-2 cells	BEA; 24, 48 and 72 h	MTT assay	Cytotoxicity	[80]
Caco-2 cells	1–20 μM BEA; 24–48–72 h	MTT assay	Cytotoxicity: IC_50_ = 4.87 ± 0.42 (24 h), 4.07 ± 0.38 (48 h), 3.16 ± 0.45 (72 h) μM	[82]
Caco-2 cells	3.125–25 μM BEA; 24–48–72 h	MTT and NR assays	MTT assay: IC_50_ = 3.2 (72 h)–20.6 (24 h) μM NR assay: IC_50_ = 1.9 (72 h)–8.8 (24 h) μM	[36]
Caco-2 cells	1.5 and 3.0 μM BEA; 4 h	LC analysis	Bioavailability: 54.3% (transport profile with 1.5 μM BEA) and 50.1% (3 μM BEA)	[79]
Coculture Caco-2 and RAW 264.7 cells	Noncytotoxic doses of BEA + silibinin; 4 h	U-HPLC-MS	Transepithelial transport of BEA: Ap = 10.40 ± 3.89% of the initial concentration; Caco-2 cells = 10.36 ± 0.98%; Bl = 2.31 ± 0.24%; Raw 264.7 cells = 0.57 ± 0.03	[78]
Caco-2 cells	1.5 μM BEA; 24 h	TEER evaluation	No barrier impairment	[83]
Caco-2 cells	1.5 μM BEA + 1.5 μM AFB1; 24 h	TEER evaluation	TEER decrease (at 1 and 2 h of exposure)	[83]
Caco-2 cells	1.5 μM BEA; 24 h	IL-8 determination	No effect on IL-8 release	[83]
Caco-2 cells	1.5 μM BEA + 3.5 μM DON; 24 h	IL-8 determination	Increased IL-8 production	[83]
Caco-2 cells	1.5 and 3.0 μM BEA; 0–120 min	DCFH-DA assay	Increased ROS production (up to 2-fold higher than control)	[36]
Caco-2 cells	1.5 and 3.0 μM BEA; 24 h	Lipid peroxidation assay	Increased MDA production (120% with 1.5 μM BEA and 207% with 3.0 μM BEA)	[36]
Caco-2 cells	1.5 and 3.0 μM BEA; 24 h	Reduced and oxidized glutathione (GSH, GSSG) determination (fluorometric method)	Dose-dependent decrease of intracellular GSH level (by 23% with 1.5 μM BEA and 31% with 3 μM BEA) Increased GSSG levels after exposure to 3 μM BEA (by 20%)	[36]
Caco-2 cells	1.5 and 3.0 μM BEA; 24, 48 and 72 h	Tetramethylrhodamine methyl ester (TMRM) method + flow cytometry	Loss of mitochondrial membrane potential (from 2% to 95% with 1.5 μM BEA and from 10% to 80% with 3.0 μM BEA)	[36]
Caco-2 cells	1.5, 3.0 and 12.0 μM BEA; 24 h	Alkaline comet assay	No increase in the TM parameter with 1.5 and 3 μM BEA; significant (*p ≤* 0.000) increase in the TM with 12.0 μM BEA	[36]
Caco-2 cells	1.5 and 3.0 μM BEA; 24, 48 and 72 h	Cell cycle analysis via propidium iodide (PI) staining	Significant (*p* ≤ 0.05) percentage of cell reduction in G0/G1 phase Increase of G2/M phase percentage	[36]
Caco-2 cells	5 μM BEA + 2.5–5 × 10^5^ CFU/mL *L. acidophilus*; 12–24–48–72 h	MTT assay	At 12 and 24 h cell viability in the presence of *L. acidophilus* and BEA is higher (*p* < 0.05) than with BEA alone	[82]
Probiotic bacterial strains typical of mammalian intestinal tract	5 mg/L BEA + 10^8^ CFU/mL; 4–48 h	LC-MS/MS	Bacteria reduced (66.5–83.1%) levels of BEA in the fermentation medium Part of the BEA was found adsorbed on the bacterial wall (2.5–8.7%) Part of BEA was internalized into the bacterial cell (42.0–79.8%) BEA interacted with components of the bacterial wall	[92]
Panel of bacterial strains typical of mammalian intestinal tract	0.1–25 μg BEA	Agar diffusion assay	Inhibition of *B. pumilus*, *B. cereus*, *B. mycoides*, *B. sphaericus*, *P. alvei*, *P. azotofixans*, *P. macquariensis*, *P. pulvifaciens* and *P. validus*. BEA also inhibited anaerobes: *E. biforme*, *P. anaerobius*, *P. productus*, *B. adolescentis* and *C. perfringens*	[89]
Panel of Gram-positive and Gram-negative bacteria	0–100 μM BEA; 18–24 h	Antimicrobial activity assay	Antibacterial activity vs. Gram-positive bacteria ((BEA) > 6 and ≤ 12.5 μM) and mycobacteria ((BEA) = 25 μM)	[81]
HT-29 cells	0–30 μM BEA; 24 and 48 h	MTT assay	Cytotoxicity (IC_50_ at 24 h = 15.00 ± 6.9 μM; IC_50_ at 48 h = 9.75 ± 4.4 μM)	[79]
N87 (human gastric cell line)	0–100 μM; 48 h	Resazurin-based in vitro toxicity assay	Cytotoxicity IC_50_ = 27.5 ± 0.7 μM)	[81]

**Table 2:** BEA bioaccessibility studies performed on a human simulated gastrointestinal environment.

Models/Cells	Concentration and Exposure Time	Method/Assay	Effects	Ref.
Simulated gastrointestinal environment	5 and 25 mg/kg BEA in the model solution and in wheat crispy bread with different natural binding compounds (dietary fibers, 1–5% *w*/*w*)	LC-MS	Bioaccessibility in the model solution: 31.8% (duodenal digestion)–54.0% (samples that underwent also colonic fermentation) Bioaccessibility in wheat crispy bread: 1.9% (duodenal digestion)–27.0% (samples that underwent also colonic fermentation)	[94]
Pro- and prebiotics in a simulated gastrointestinal environment	Model solution: 10 mg/L BEA + 2 × 10^6^ CFU and 10 mg/L BEA + 1–5 g dietary fibers	LC-MS	Probiotics and prebiotics caused a reduction of BEA bioaccessibility (30–85% and 60–80%, respectively) Identification of a BEA degradation product by colonic fermentation	[96]
Pro- and prebiotics in dynamic simulated gastrointestinal environment	BEA in 20 g of wheat crispy bread produced with 300 g of wheat flour added with 10^6^ conidia/mL of *F. tricinctum*, *F. culmorum*, *G. zeae* and fermented for 30 days + 1–5% *w*/*w* prebiotics or 1 × 10^5^ UFC/mL probiotics in the simulated saliva	LC-MS/MS	Probiotics (highest reduction 10.1 and 6.6%, gastric and duodenal bioaccessibility, respectively, ctr 15.8 and 11.3%) and prebiotics (highest reduction 15.3 and 12.0%, gastric and duodenal bioaccessibility, respectively, ctr 28.4 and 19.6%) caused a reduction of BEA bioaccessibility	[97]
Static and dynamic simulated gastrointestinal environment	BEA in 10 g (static model) or 100 g (dynamic model) of wheat crispy bread produced with 300 g of durum wheat added with 10^6^ conidia/mL of *F. tricinctum* and fermented for 30 days, mixed with 1–5% *w*/*w* inulin and fructooligosaccharides (FOS)	LC-MS/MS	BEA bioaccessibility in the static model: 46.7–61.1%; BEA bioaccessibility in the dynamic model: 76.2–91.0% The addition of inulin and FOS decreased (−23.9%) BEA bioaccessibility	[95]

**Table 3:** In vitro effects of BEA on species-specific intestinal cell models.

Models/Cells	Concentration and Exposure Time	Method/Assay	Effects	Ref.
Proliferating IPEC-J2 cells	0–10 μM BEA; 24 h	Cytotoxicity assay (flow cytometry + Annexin-V-FITC and PI staining)	5 μM BEA: 82% viable, 15% early apoptotic, 3% apoptotic/necrotic cells; 10 μM BEA complete disruption	[50]
Differentiated IPEC-J2 cells	0–10 μM BEA; 24 h	Cytotoxicity assay (flow cytometry + Annexin-V-FITC and PI staining)	5 μM BEA no effect; 10 μM BEA: 47% viable, 27% early apoptotic, 27% apoptotic/necrotic cells	[50]
IPEC-J2 cells	1.5–10 μM BEA; 24, 48 and 72 h	TEER evaluation	5 μM BEA significantly (*p* < 0.05) reduced TEER starting from 24 h of exposure; 80% reduction after 72 h of exposure to 10 μM BEA	[103]
IPEC-J2 cells	1.5–10 μM BEA; 24, 48 and 72 h	NR assay	No cytotoxicity	[103]
IPEC-J2 cells	2.5 μM BEA + 1.5 or 3 μM DON; 24, 48 and 72 h	TEER evaluation	No effect on barrier integrity	[103]
IPEC-J2 cells	2.5 μM BEA + 1.5 or 3 μM DON; 24, 48 and 72 h	NR assay	No cytotoxicity	[103]
IPEC-J2 cells	0–20 μM BEA; 48 h	Sulforhodamine B (SRB) assay	Cytotoxicity: absolute IC_50_ = 2.43 μM; relative IC_50_ = 2.24 μM	[101]
IPEC-1 cells	BEA; 48 h	CellTiter-Glo^®^ Luminescent Cell Viability Assay	Cytotoxicity: IC_50_ = 4.3 ± 1.8 μM (classified as highly toxic)	[102]

**Table 4:** In vitro effects of enniatins (ENNs) on human intestinal cell models.

Models/Cells	Concentration and Exposure Time	Method/Assay	Effects	Ref.
Caco-2 cells	0–100 μM ENN; 48 h	Resazurin-based in vitro toxicity assay	Cytotoxicity IC_50_ range = (1.1 ± 0.2 μM (ENN A))–(4.6 ± 1.3 μM (ENN B))	[81]
Caco-2 cells	0.1–10 μM ENN; 72 h	MTT assay	IC_75_ = 1.38 ± 0.07 μM; IC_50_ = 1.99 ± 0.09 μM; IC_25_ = 2.63 ± 0.21 μM	[123]
Caco-2 cells	ENN; 24, 48 and 72 h	MTT assay	Cytotoxicity (3- to 4-fold higher than BEA)	[80]
Caco-2 cells	1–25 μM ENN B; 24 h	Cell counting	Viability: 85 ± 7% after exposure to 1 μM ENN B; 50 ± 5% after exposure to 25 μM ENN B	[113]
Caco-2 cells (undifferentiated and differentiated)	0.6–30 μM ENN B; 24 and 48 h	MTT assay	Decreased viability after exposure to concentrations ≥6 μM (both undifferentiated and differentiated cells) IC_50_ value not reached in the range of concentrations and time of exposure tested (both undifferentiated and differentiated cells)	[125]
Caco-2 cells	Up to 437.2 μM ENN B; max 48 h	Combined bioassays for cytotoxicity (AB –metabolic activity, LDH—cell membrane integrity and NR—lysosomal activity	Significant cytotoxicity observed only in connection with lysosomal functionality, EC_50_ at 3 h = 10 ± 3.8 μM; EC_50_ at 24 h = 2.1 ± 0.4 μM When performed in presence of the antioxidant ascorbic acid, NR assay revealed no reduction of the ENN B-induced cytotoxicity No effect of ENN B on AB and LDH assay	[113]
Caco-2 cells (undifferentiated and differentiated)	ENN J3	Cytotoxicity assay	No cytotoxic effects at any of the concentrations tested	[145]
Caco-2 cells	0.6–30 μM ENN B, B1, A, A1; 24 h	MTT assay	IC_50_ ENN A1 and B1 = 2.3 and 19.5 μM, respectively ENN A and B: no cytotoxic effect at the tested concentrations	[144]
Caco-2 cells	0–30 μM ENN A, A1, B, B1; 24 h	MTT assay	IC_50_ A1 = 12.3 μM; IC_50_ B1 = 19.5 μM. No IC_50_ were obtained for ENN A and B at the concentrations tested	[141]
Caco-2 cells	0.6-30 μM ENN A, A1, A2, B, B1, B4 and J3; 24 and 48 h	MTT assay	At 48 h (μM): IC_50_ A = 9.3 ± 0.6; IC_50_ A1 = 2.7 ± 0.8; IC_50_ A2 = 2.6 ± 0.7; IC_50_ B = no values obtained in the range of concentrations tested; IC_50_ B1 = 11.5 ± 5.3; IC_50_ B4 = 4.5 ± 2.9 and IC_50_ J3= no values obtained in the range of concentrations tested	[120]
Caco-2 cells	0–50 nM ENNs (A, A1, B, B1); 72 h	Resazurin-based in vitro toxicity assay	No cytotoxicity	[78]
Caco-2 cells	0.9–15 μM ENN A1, B, B1 and 0.45–7.5 μM ENN A; 24, 48, 72 h	MTT and NR assay	MTT assay, at 72 h (μM): IC_50_ A = 1.6 ± 0.8; IC_50_ A1 = 1.3 ± 0.6; IC_50_ B = 11.7 ± 2.4; IC_50_ B1 = 2.8 ± 1.1. NR assay, at 72 h (μM): IC_50_ A = 0.46 ± 0.1; IC_50_ A1 = 0.46 ± 0.1; IC_50_ B = 1.4 ± 0.2; IC_50_ B1 = 0.8 ± 0.3	[116]
Caco-2 cells	0.9–15.0 μM ENN A, A1, B, B1 alone and combined; 24 h	MTT assay	Dose-dependent cytotoxic effects (individually and in combination) Synergistic effect observed for ENN B + ENN A1, ENN B1 + ENN A1 and ENN A + ENN A1 + ENN B Additive effect at a medium and high affected fraction for all the other combinations (exception: lower fraction affected and ENN B + ENN B1 that produced antagonistic effects)	[122]
Caco-2 cells	0.312–10 μM ENN B (alone), 0.312–5 μM in coexposures; 24, 48 and 72 h	MTT assay	IC_50_ ENN B = 3.87 μM (at 72 h) At 72 h: IC_50_ ENN B + DON = 1.71 ± 0.61 μM (antagonism); IC_50_ ENN B + AOH = 0.91 ± 0.32 μM (additive effect); IC_50_ ENN B + AOH + DON = 1.63 ± 0.44 μM (antagonism)	[147]
Caco-2 cells	Binary mixture of ENN B + other *Fusarium* and *Alternaria* mycotoxins (0–250 μM); 24 h	Cell proliferation assay WST-1	ENN B was the most cytotoxic compound (IC_50_ = 6.3 μM) Binary mixtures (at cytotoxic concentrations) of ENN B + deoxynivalenol, nivalenol, zearalenone and tenuazonic acid produced antagonistic effects	[146]
Caco-2 cells	1–25 μM ENN B; 24 h	Double staining with PI/Hoechst 33342 and fluorescence microscopy	Dose-dependent increase of necrotic cells (13% after25 μM ENN B for 24 h) with a corresponding decrease in the number of normal cells Max 2% of apoptotic cells (1 μM ENN B), with a decrease at higher concentrations	[113]
Caco-2 cells	1.5 and 3 μM ENN A, A1, B and B1; 24, 48 and 72 h	Flow cytometry analysis of apoptosis and necrosis (V-FITC/PI double staining)	Apoptotic effects observed after 24 and 48 h of exposure; necrotic effects observed for ENN A and A1 after 24 h, and for all mycotoxins at 72 h	[116]
Caco-2 cells	ENN B1	Absorption profile	Permeability of ENN B1 Bl to Ap is 6.7-times higher than in the opposite direction Ap to Bl transport of ENN B1 increased significantly after treatment with Pgp (verapamil) and MRP2 (MK571) inhibitors No significant effect on Ap to Bl ENN B1 transport after treatment with BCRP inhibitor (fumitremorgin C)	[140]
Caco-2 cells	1.5 and 3.0 μM (ENN A, A1, B and B1); 1–4 h	LC-DAD and LC-MS	Transport profile at 4 h (1.5 and 3.0 μM): ENN A = 76.8 ± 1.3% and 57.7 ± 1.2%; ENN A1 = 70.2 ± 1.2% and 68.8 ± 0.8%; ENN B = 67.0 ± 0.7% and 65.0 ± 1.2%; ENN B1 = 62.2 ± 1.2% and 65.1 ± 1.1% Duodenal bioavailability: between 148.5 ± 4.2% (3.0 μM ENN A) and 197.1 ± 4.2% (1.5 μM ENN A)	[142]
Caco-2 cells	ENNs in the duodenal fluid from the simulated gastrointestinal digestion of 3 g of wheat crispy bread spiked with ENN A, A1, B and B1 at 1.5 and 3.0 μmol/g (1.5 and 3.0 μM)	LC-DAD	Transport profile at 4 h (1.5 and 3.0 μmol/g): ENN A = 70.8 ± 1.3% and 50.7 ± 1.3%; ENN A1 = 64.2 ± 1.0% and 73.8 ± 0.9%; ENN B = 64.0 ± 0.9% and 59.0 ± 1.2%; ENN B1 = 55.2 ± 1.1% and 66.1 ± 1.0% ENN bioavailabilities between 40.8 ± 1.0% (ENN B) and 60.0 ± 1.0% (ENN A)	[141]
Caco-2 cells grown with bacterial strains (10^7^–10^8^ CFU/mL) typical of the intestinal tract	1.5 and 3.0 μM (ENN A, A1, B and B1); 48 h	LC-MS	Colonic transport: highest concentration of ENNs in the Bl side (from 0.10 ± 0.05 μM -ENN A to 0.54 ± 0.08 μM -ENN B1), followed by the cell matrix (from 0.10 ± 0.07 μM -ENN A1 to 0.68 ± 0.10 μM -ENN A) and Ap side (from 0.05 ± 0.01 μM -ENN B1 to 0.30 ± 0.07 μM -ENN A) Colonic bioavailability: between 17.32 ± 2.83% (ENN A) and 57.45 ± 3.12% (ENN B1)	[142]
Caco-2 cells	1–25 μM ENN B; 24 h	PI staining and flow cytometry	Increase of the cells in the G2/M phase (25 μM ENN B for 24 h = 31 ± 1.3% cells in the G2/M phase; control = 23 ± 1.0%)	[113]
Caco-2 cells	1–25 μM ENN B; 24 h	Red fluorescent LysoTracker Red DND-99 staining	Fluorescent intensities strongly decreased after 24 h exposure Rupture of lysosomal membranes after 3 h of exposure to 10 μM ENN B	[113]
Caco-2 cells	5 and 10 μM ENN B; 3 and 24 h	Lipophilic cationic probe + flow cytometry	Dose-dependent decrease in the FL2/FL1 ratio after exposure to 5 μM and 10 μM ENN B for 24 h (indicator of change in the membrane potential and MOMP)	[113]
Caco-2 cells	1–25 μM ENN B; 3 and 24 h	Dihydroethidium (DHE) (oxidation- sensitive fluorescent probe for ROS detection)	Increased ROS production already with 5 μM ENN B for 3 h; 2.4-fold increase after 24 h	[113]
Caco-2 cells	1.5 and 3 μM ENN A, A1, B and B1; up to 120 min	ROS generation assay (H_2_-DCFDA probe)	ROS production depends on ENN concentration and time of exposure ROS production observed at the early stage of exposures (ENN A1, B and B1) Higher ROS production (2.1-times the control) with 3.0 μM ENN B1 exposure from 5 to 120 min	[116]
Caco-2 cells	1.5 and 3 μM ENN A, A1, B and B1; 24 h	Lipid peroxidation assay (TBARS method)	Increased MDA production: at 3 μM, increase of 111% (ENN A), 58% (ENNA1), 48% (ENN B), 59% (ENN B1)	[116]
Caco-2 cells	1.5 and 3 μM ENN A, A1, B and B1; 24, 48 and 72 h	Cell cycle analysis (PI staining) by flow cytometry	Alteration (time- and concentration-dependent) of the % of cells in SubG0/G1, G0/G1 and G2/M phases for all ENNs tested	[116]
n	1.5 and 3 μM ENN A, A1, B and B1; 24, 48 and 72 h	Detection of mitochondrial membrane potential by tetramethyl rhodamine methyl ester (TMRM) method	Dose- and time-dependent decrease in TMRM fluorescence intensity. At 72 h reduction, % of TMRM intensity ranged from 91.0% (ENN B) to 98.7% (ENN A)	[116]
Caco-2 cells	1.5 and 3 μM ENN A, A1, B and B1; 24 h	Alkaline comet assay	ENN A (1.5 and 3.0 μM) and 3.0 μM ENN A1 and B1 induced a significant increase in the TM parameter; 1.5 μM ENN A1 and ENN B did not cause any DNA damage	[116]
Coculture Caco-2 and RAW 264.7 cells	Noncytotoxic doses of ENNs (A, A1, B, B1) + silibinin; 4 h	U-HPLC-MS	Transepithelial transport of ENNs (ranges): Ap = (9.52 ± 3.02% (ENN A1))–(24.08 ± 3.20% (ENN B)) of the initial concentration; Caco-2 cells = (0.51 ± 0.03% (ENN B1))–(10.76 ± 0.43% (ENN A)); Bl = (13.06 ± 0.45% (ENN A1))–(28.16 ± 3.37% (ENN B1)); Raw 264.7 cells = (0.22 ± 0.03% (ENN A1))–(0.60 ± 0.10% (ENN B1))	[78]
Panel of Gram-positive and Gram-negative bacteria	0–100 μM ENNs; 18–24 h	Antimicrobial activity assay	Antibacterial activity vs. Gram-positive bacteria (range: 3.12 μM (ENN A)–>100 μM (ENN B)) and mycobacteria (range: 3.12 μM (ENN A1)–100 μM (ENN B))	[81]
Bacterial strains normal pathogens of the intestinal tract	0.2–2000 μg ENN B; 48 h	Disk diffusion assay	ROS production depends on ENN concentration and time of exposureInhibition of several micro-organisms (major inhibitory effects on *C. perfringens* and *S. aureus* CECT 976) No effects on *S. aureus* CECT 240, *E. coli* CECT 4782 and *S. dysenteriae* CECT 584	[125]
Probiotic bacteria, *Saccharomyces cerevisiae* strains and *Bacillus subtilis* strains	0.2–20,000 ng ENN A, A1, A2, B, B1 and B4; 24 h	Disk diffusion assay	ENNs active against many micro-organisms (inhibition halos 3-12 mm) ENN A1 most active compound (reduction of the growth of 8 strains at a dose of 20,000 ng), followed by ENN B1 (inhibition of 6 strains) while ENN B and B4 had no antimicrobial effects at concentrations up to 20,000 ng per disk.	[127]
HCT116 cells	0–10 μM ENN; 24 h	^3^H-thymidine incorporation	p53-dependent cytostatic and p53-independent cytotoxic activities	[123]
HT29	0.6–30 μM ENN A, A1, A2, B, B1, B4 and J3; 24 and 48 h	MTT assay	At 48 h (μM): IC_50_ A = 8.2 ± 1.8; IC_50_ A1 = 1.4 ± 0.7; IC_50_ A2 = no values obtained in the range of concentrations tested; IC_50_ B = 2.8 ± 0.9; IC_50_ B1 = 3.7 ± 0.7; IC_50_ B4 = 15.0 ± 4.0 and IC_50_ J3 = no values obtained in the range of concentrations tested	[120]
N87 (human gastric cell line)	0–100 μM ENN; 48 h	Resazurin-based in vitro toxicity assay	Cytotoxicity IC_50_ range = (0.003 ± 0.002 μM (ENN A1))–(1.7 ± 0.1 μM (ENN B))	[81]
ACD/Percepta (in silico model)	ENNs (A, A1, B, B1)	In silico toxicity analysis	Very low aqueous solubility and low bioavailability No genotoxicity Caco-2 permeability (Pe) = 6.0-6.1 10^−4^ cm/s Weaker substrate than BEA for Pgp	[78]

**Table 5:** ENN bioaccessibility studies performed on a human simulated gastrointestinal environment.

Models/Cells	Concentration and Exposure Time	Method/Assay	Effects	Ref.
Simulated gastrointestinal environment	ENN A, A1, B, B1 spiked (1.5 and 3.0 μmol/g) in wheat crispy bread (sample: 3 g)	LC-DAD	Duodenal bioaccessibility (1.5 and 3.0 μmol/g) ENN A = 84.6 ± 2.5% and 87.3 ± 2.9%; ENN A1 = 72.6 ± 1.8% and 70.0 ± 1.7%; ENN B = 68.6 ± 2.9% and 73.3 ± 1.5%; ENN B1 = 74.0 ± 1.6; 74.0 ± 1.9%	[141]
Simulated gastrointestinal environment	ENN A, A1, B, B1 added (1.5 and 3.0 μmol/g) to breakfast cereals, cookies and breads (sample: 3 g)	LC-DAD	Lowest bioaccessibility values found in wheat bran with fibers (1.5 μM ENN A = 50.1 ± 3.1%; 1.5 μM ENN A1 = 40.4 ± 1.9%; 1.5 μM ENN B = 43.9 ± 3.4%; 1.5 μM ENN B1 = 46.3 ± 3.1%); highest values in white loaf bread (1.5 μM ENN A = 79.9 ± 2.8%; 1.5 μM ENN A1 = 64.2 ± 2.4%; 1.5 μM ENN B = 69.8 ± 2.9%; 1.5 μM ENN B1 = 73.6 ± 2.2%)	[149]
Simulated gastrointestinal environment	ENN A, A1, B, B1 spiked (1.5 and 3.0 μmol/g) in 3 g of wheat crispy bread with 0, 1, 5 or 10% inulin	LC-DAD	Bioaccessibility of ENNs in crispy bread without inulin: 69% (1.5 μM ENN B) and 87% (3.0 μM ENN A); bioaccessibility with 1% inulin: 65% (3 μM ENN A)–83% (1.5 μM ENN A); bioaccessibility with 5% inulin: 58% (3.0 μM ENN A1)–74% (1.5 μM ENN B1); bioaccessibility with 10% inulin 51% (3 μM ENN A)–74% (1.5 μM ENN B1)	[148]
Simulated gastrointestinal environment	ENNs (A, A1, B and B1) in follow-up infant formula	LC-DAD	Colonic + duodenal bioaccessibility, range: ENN A = 1.63 ± 0.01% (just one sample found positive); ENN A1 = (4.36 ± 0.61%)–(60.53 ± 2.64%); ENN B1 = (1.49 ± 0.04%)–(8.43 ± 0.64%). No samples found positive for ENN B	[151]
Static and dynamic simulated gastrointestinal environment	ENNs in 10 g (static model) or 100 g (dynamic model) of wheat crispy bread produced with 300 g of durum wheat added with 10^6^ conidia/mL of *F. tricinctum* and fermented for 30 days, mixed with 1–5% w/w inulin and fructooligosaccharides (FOS)	LC-MS/MS	ENN bioaccessibility in the static model: 6.2–44.9%; ENN bioaccessibility in the dynamic model: 23.0–68.9% ENN bioaccessibility of the inulin-enriched samples (1 and 5%) were 32.7 and 23.0%, respectively (29.0–50.5% lower than the control). Similar results obtained with FOS	[95]
Pro- and prebiotics in dynamic simulated gastrointestinal environment	ENNs (A, A1, B and B1) in 20 g of wheat crispy bread produced with 300 g of wheat flour added with 10^6^ conidia/mL of *F. tricinctum*, *F. culmorum*, *G. zeae* and fermented for 30 days + 1–5% *w*/*w* prebiotics or 1 × 10^5^ UFC/mL probiotics in the simulated saliva	LC-MS/MS	Probiotics (highest reduction 27.8 and 21.2%, gastric and duodenal bioaccessibility, respectively, ctr 39.6 and 33.4%) and prebiotics/food ingredients (highest reduction 20.3 and 17.0%, gastric and duodenal bioaccessibility, respectively, ctr 39.6 and 33.4%) caused a reduction of ENNs bioaccessibility	[97]
Simulated gastrointestinal environment	500 μg/L ENN B (final concentration in the digestion solution)	Targeted and untargeted UHPLC-MS/MS	ENN B overall degradation rate = 78.45 ± 5.39% (low stability in the in vitro gastrointestinal condition Identification of a total of 5 catabolic metabolites plus isomers (M1–M5) Putative metabolites subjected to in silico pharmacokinetic evaluation with BOILED-Egg predictive model (M4 and M5 are probably not absorbed at gastrointestinal level; M2 and ENN B likely absorbed; M1 and M3 showed possible moderate crossing of the blood–brain barrier where no interaction with Pgp was predicted; M1, M2, M4 and M5 are likely CYP2C19 inhibitors but none of the metabolites is expected to inhibit CYP1A2, CYP3A4, CYP2D6 or CYP2C9)	[105]

**Table 6:** In vitro effects of ENNs on species-specific intestinal cell models.

Models/Cells	Concentration and Exposure Time	Method/Assay	Effects	Ref.
IPEC-1 cells	0–100 μM ENN alone and in coexposure with 0–100 nM T2; 48 h	CellTiter- Glo^®^ Luminescent Cell Viability Assay	IC_50_ ENN = 15.80 μM; IC_50_ T2 = 9.35 nM; IC_50_ T2 + ENN (1:1000) = 14.41 μM	[153]
IPEC-1 cells	ENN A1, B, B1; 48 h	CellTiter-Glo^®^ Luminescent Cell Viability Assay	IC_50_ ENN A1 = 1.6 ± 0.3 μM (classified as highly toxic); IC_50_ ENN B = 4.4 ± 0.9 μM (highly toxic); IC_50_ ENN B1 = 13.5 ± 2.5 μM (moderately toxic)	[102]
Proliferating IPEC-J2 cells	0–100 μM ENN A, A1, B, B1; 24 h	Cytotoxicity assay (flow cytometry + Annexin-V-FITC and PI staining)	5 μM ENNs: no effects; ENN A most cytotoxic (10 μM exposure caused a reduction of viable cells to 30%); 10 μM ENN A1 and ENN B1: 86% and 93% viable cells, respectively while 25 μM ENN A1 and ENN B1: complete disruption and 25% viable cells, respectively; 25 μM ENN B: 92% viable cells 5 μM ENN A and ENN A1: 4.1% and 3.5% early apoptosis and 2.7% and 2.4% late apoptosis/necrosis, respectively	[50]
Differentiated IPEC-J2 cells	0–100 μM ENN A, A1, B, B1; 24 h	Cytotoxicity assay (flow cytometry + Annexin-V-FITC and PI staining)	5 μM ENNs no effect; ENN A most cytotoxic (10 μM exposure caused a reduction of viable cells to 36%); 25 μM ENN A1 and ENN B1: complete disruption and 36% viable cells, respectively; 25 μM ENN B: 89% viable cells	[50]
IPEC-J2 cells	0–20 μM ENN A, A1, B, B1; 48 h	Sulforhodamine B (SRB) assay	Cytotoxicity: IC_50_ ENN A = 3.40 μM; IC_50_ ENN A1 = 4.15 μM; IC_50_ ENN B = 3.25 μM; IC_50_ ENN B1 = 3.67 μM	[101]
IPEC-J2 cells	0–10 μM ENNs (A, A1, B and B1); 24, 48 and 72 h	TEER evaluation	ENN A: TEER reduction after 72 h exposure to 5 μM; ENN A1: TEER reduction starting from 24 h exposure to 10 μM; ENN B: TEER reduction after 48 h and 72 h of exposure to 2.5 and 5 μM; ENN B1: TEER reduction starting from 48 h of exposure to 5 μM;	[103]
IPEC-J2 cells	1.5–3 μM ENNs (A, A1, B and B1) in absence or presence of 1.5-3 μM DON; 24, 48 and 72 h	TEER evaluation	ENN A + A1 + B + B1: TEER reduction starting from 24 h exposure to 1.5 μM; ENN A + A1 + B + B1 + DON: TEER reduction starting from 24 h exposure to 1.5 μM	[103]
IPEC-J2 cells	0–10 μM ENNs (A, A1, B and B1); 72 h	NR assay	No cytotoxicity	[103]
IPEC-J2 cells	1.5–3 μM ENNs (A, A1, B and B1) in absence or presence of 1.5–3 μM DON; 72 h	NR assay	No cytotoxicity	[103]
Calf small intestinal epithelial cells B	0–200 μM ENN B; 48 h	NR assay and WST-1 assay	IC_50_ ENN B = 4.0–6.7 μM	[154]

**Table 7:** ENN bioaccessibility studies performed on rumen models.

Models/Cells	Concentration and Exposure Time	Method/Assay	Effects	Ref.
In vitro rumen model	50 mg of maize silage spiked with 1 mg/Kg ENN B; up to 48 h	UPLC system coupled to Xevo^®^ TQ-S MS/MS system	ENN B degradation up to 72% (48 h of incubation)	[159]
In vitro rumen model	50 mg of maize silage spiked with 1 mg/Kg ENN B + 3 g/kg Binder 1, 2 and 3; up to 48 h	UPLC system coupled to Xevo^®^ TQ-S MS/MS system	Binder 1 (clay minerals and yeast derivatives): adsorbed ENN B by 24%; Binder 2 (bentonite, leonardite, plant extracts, epoxidase): adsorbed ENN B by 28%; Binder 3 (bentonite and sepiolite): adsorbed ENN B by 22%	[160]

**Table 8:** Examples of the occurrence of BEA and ENNs in food chain products: number of tested samples and maximum found concentrations (μg/kg).

Source	Tested Samples	BEA	ENN A	ENN A1	ENN B	ENN B1	Country	References
Rice Flour	93	810.1	<LOQ	<LOQ	<LOQ	2.4	Brazil	[161]
Rice Husk	93	110.4	<LOQ	<LOQ	2.6	1.2	Brazil	[161]
Wheat	97	9.1	139.8	356.0	814.6	510.0	Romania	[162]
Wheat	140	13.5	15.6	165.0	2168	776.7	Belgium	[163]
Barley	10	ND	ND	ND	1.3	ND	Spain	[10]
Rice bran	4	64.8	ND	ND	ND	ND	Spain	[10]
Corn pulp	4	37.8	ND	ND	2.2	ND	Spain	[10]
Barley hulless	12	423	13	87	592	281	Czech Republic	[167]
Oats hulless	12	35	ND	ND	55	15	Czech Republic	[167]
Barley	56	130	39	140	2100	520	Denmark	[27]
Oat	11	110	<10	39	470	120	Denmark	[27]
Wheat	33	<15	<10	60	1600	290	Denmark	[27]
Rye	10	<15	100	100	3900	860	Denmark	[27]
Spring barley	8	ND	3	45	301	240	Poland	[168]
Winter barley	16	ND	ND	16	253	81	Poland	[168]
Oats	4	ND	ND	11	162	67	Poland	[168]
Triticale	20	ND	135	882	3328	1347	Poland	[168]
Maize	73	136	17.1	27.4	1.52	16.3	Serbia	[26]
Sugar beet pulp	1	3.0	ND	ND	ND	ND	Spain	[10]
Silage (maize, grass)	120	228	9.14	51.2	101	57.2	Poland	[169]
Formula feed								
Pig	1141	413	307	549	1514	1846	Austria	[101]
Pig	228	747	64.9	140	1222	247	Spain	[170]
Poultry	78	474.9	34.7	32.1	2190.2	396.0	UK	[165]
Bovine	8	51.4	ND	10.7	41.6	20.2	Spain	[10]
Ovine	13	129.6	ND	13.1	89.5	28.8	Spain	[10]
Caprine	1	23.2	ND	8.5	23.9	15	Spain	[10]
Horses	3	29.8	ND	10.1	43.8	15.5	Spain	[10]
Porcine	4	14.6	ND	11.9	55.1	24.0	Spain	[10]
Poultry	11	23.8	ND	11.9	51.1	23.1	Spain	[10]
Rabbits	2	13.5	ND	11.8	50.3	23.6	Spain	[10]
Dogs	3	40.5	ND	ND	24.8	10.1	Spain	[10]
Cats	3	ND	ND	ND	6.7	8.9	Spain	[10]
Chicken	43	29.3	12.6	5	39.8	15.7	Tunisia	[171]
Cattle	35	5.7	0.9	4.3	21.9	ND	Tunisia	[171]
Rabbit	12	2.3	ND	7.9	9.7	26.6	Tunisia	[171]
Sheep	16	ND	4.3	20.5	21.7	12.8	Tunisia	[171]
Horse	16	2.1	ND	ND	2.4	1.5	Tunisia	[171]
Human								
Breast milk	87	0.0017	<LOQ	<LOQ	0.0086	0.0019	Austria	[172]
Fish								
Sea bass (*Dicentrarchus labrax*)	10	ND or <LOQ	ND or <LOQ	6.9	12.8	31.5	Spain (from aquaculture)	[112]
Sea bream (*Sparus aurata*)	10	ND or <LOQ	ND or <LOQ	7.5	21.6	19	Greece (from aquaculture)	[112]
Atlantic salmon (*Salmo salar*)	10	ND or <LOQ	ND or <LOQ	29	103	94	Norway (from aquaculture)	[112]
Rainbow trout (*Oncorhynchus mykiss*)	10	ND or <LOQ	ND or <LOQ	ND	3.6	2.9	Spain (from aquaculture)	[112]

ND = not determined or not detected; LOQ = below the limit of quantification
